# Assessing the Risk for Resistance and Elucidating the Genetics of *Colletotrichum truncatum* That Is Only Sensitive to Some DMI Fungicides

**DOI:** 10.3389/fmicb.2017.01779

**Published:** 2017-09-15

**Authors:** Can Zhang, Yongzhao Diao, Weizhen Wang, Jianjun Hao, Muhammad Imran, Hongxia Duan, Xili Liu

**Affiliations:** ^1^Department of Plant Pathology, China Agricultural University Beijing, China; ^2^State Key Laboratory of Mycology, Institute of Microbiology, Chinese Academy of Sciences Beijing, China; ^3^School of Food and Agriculture, University of Maine, Orono ME, United States; ^4^Department of Applied Chemistry, China Agricultural University Beijing, China

**Keywords:** *Colletotrichum truncatum*, DMI fungicides, mechanism of sensitivity differentiation, M376L/H373N, resistance analysis

## Abstract

The genus *Colletotrichum* contains a wide variety of important plant pathogens, and *Colletotrichum truncatum* is one of the most prevalent species of *Colletotrichum* on chili in China. Demethylation-inhibitor fungicides (DMIs) are currently registered chemical agents for the management of the anthracnose disease caused by *Colletotrichum* spp. To assess the risk for DMI resistance development, 112 *C. truncatum* isolates were collected from infected pepper in 13 regions of China. The sensitivity of *C. truncatum* isolates to five DMI fungicides was determined based on mycelial growth inhibition assay. *C. truncatum* was sensitive to prochloraz, epoxiconazole, and difenoconazole, but not to tebuconazole or myclobutanil. Baseline sensitivity using the 112 *C. truncatum* isolates was established for the first three effective DMIs. Prochloraz, epoxiconazole, and difenoconazole EC_50_ values were 0.053 ± 0.023, 1.956 ± 0.815, and 1.027 ± 0.644 μg/ml, respectively. Eleven stable DMI-resistant mutants all exhibited lower fitness levels than their wild-type parents, suggesting a low risk of DMI resistance in *C. truncatum*. By inducing gene expression, *CtCYP51* expression increased slightly in the resistant mutants as compared to wild-types when exposed to DMI fungicides and thus contributed at least partially to resistance. Molecular docking with CYP51 structure models was used to explain differential sensitivity of the DMI fungicides in *C. truncatum*. Our results suggest that the M376L/H373N mutations in CYP51 changed the conformation of DMIs in the binding pocket. These changes prevented the formation of the Fe – N coordinate bond between the heme iron active site and tebuconazole or myclobutanil, and apparently contributed to tebuconazole and myclobutanil insensitivity of *C. truncatum*.

## Introduction

The genus *Colletotrichum* contains a wide variety of important plant pathogens (Bailey and Jeger, 1992; Cannon et al., 2012; Dean et al., 2012), which infect more than 460 woody and herbaceous plants including pepper (Shenoy et al., 2007; Damm et al., 2009; Wikee et al., 2011). Pepper (*Capsicum* spp.) is an economically important vegetable crop worldwide and China is a main producer and exporter of chili and other peppers (Valadez-Bustos et al., 2009). *C. truncatum* is the most prevalent species of *Colletotrichum* on chili in China, and annual yield losses to peppers caused by *C. truncatum* can be as high as 30–50% (Qing et al., 2005; Diao et al., 2016).

*Colletotrichum* spp. cause anthracnose on many plants. Currently the most efficient control of this disease is through the application of agrochemicals. Since the 1990s, benzimidazoles, including carbendazim and thiophanate-methyl, have been the most commonly used fungicides for anthracnose control (Xu et al., 2014). However, benzimidazole-resistant isolates of *Colletotrichum* have been commonly found in China, and failures of control have been frequently reported by growers since 2009 (Han et al., 2009; Chen et al., 2013; Yang et al., 2015). Demethylation-inhibitor fungicides (DMIs) have a broad spectrum of antifungal activity and they can move systemically in the plant (Hof, 2001). DMIs are considered to be alternatives to benzimidazole fungicides, and some of them have been registered for the control of anthracnose in China (Xu et al., 2014). DMI inhibitors that have been used as agricultural fungicides include imidazoles and triazoles. DMIs inhibit fungal growth by interacting with the heme-iron of the cytochrome P450 sterol 14α-demethylase (CYP51), an enzyme essential for the biosynthesis of the predominant sterol of fungal membranes (Köller, 1992, 1996). The DMIs are particularly useful for disease control because of their post-infection activity against fungal plant pathogens (Szkolnik, 1981).

Resistance to DMI fungicides is common for a number of fungal plant pathogens (Singh et al., 1996; O’Quinn et al., 2001; Serfling et al., 2007). To date, three mechanisms of resistance to azoles have been reported: (i) single or multiple mutational changes in the *CYP51* gene resulting in amino acid alterations that decrease the affinity between the protein and inhibitors (Cools et al., 2010; Nina et al., 2015); (ii) upregulation of the *CYP51* gene which increases target abundance and therefore dilutes DMI binding (Luo and Schnabel, 2008; Nina et al., 2015); and (iii) overexpression of genes encoding drug efflux pumps, such as ATP binding cassette (ABC) transporters, which transport inhibitors out of the cell and result in multidrug resistance (Kretschmer et al., 2009; Nina et al., 2015).

Prochloraz (an imidazole), epoxiconazole, difenoconazole, tebuconazole and myclobutanil (four triazoles) are the DMI fungicides that have been registered for the control of anthracnose since 2012 in China^1^. However, the sensitivity to different DMIs has never been determined for *C. truncatum* in China. In addition, the resistance of *C. truncatum* to DMIs has rarely been examined. In the current study, we firstly determined the baseline sensitivity of 112 wild-type *C. truncatum* isolates to five commonly used DMIs, and assessed the risk of resistance of *C. truncatum* to these fungicides. The results indicated that natural populations of *C. truncatum* were only sensitive to prochloraz, epoxiconazole and difenoconazole, but insensitive to tebuconazole and myclobutanil. Therefore, the basis of this sensitivity differentiation to DMIs in *C. truncatum* was investigated.

## Materials and Methods

### Fungicides

The information of fungicides, including the active ingredients, the distributors, and the concentrations used in this study are listed in Supplementary Table S1. For bioassays, a stock solution of 10 g/liter active ingredient of each fungicide was made in dimethyl sulfoxide (DMSO) and stored at 4°C. The stock solutions were added to molten PDA at various concentrations (Supplementary Table S1) after the medium was cooled to ≈50°C. The final concentration of the solvent DMSO was 0.1% in the medium.

### Cultivation of *C. truncatum* and Adaptation to DMIs

A total of 112 *C. truncatum* isolates were collected in 2011, 2012, and 2013 from 13 regions in China in a previous study (Diao et al., 2015); Background information of the isolates is provided in Supplementary Table S2. Based on both morphological characteristics and phylogenetic analysis, all 112 isolates were previously determined to be *C. truncatum* (Damm et al., 2009; Diao et al., 2015). Sensitivity of the 112 *C. truncatum* field isolates to prochloraz, epoxiconazole, difenoconazole, tebuconazole, and myclobutanil was determined by calculating the 50% inhibitory dose (EC_50_ value, μg/ml) on fungicide-amended PDA in Petri plates (Supplementary Table S1) as previously reported (Russell, 2004; Fan et al., 2014). Plates were incubated at 28°C for 4 days in darkness. Each combination of isolate and fungicide was represented by three replicate plates, and the experiment was performed twice.

### Generation of DMI-Resistant Mutants

All the 112 field isolates were used in the mutant selection. For each wild-type isolate, 50 agar plugs were placed on PDA containing one of the DMIs at a concentration of EC_90_. Agar plugs were placed on PDA containing prochloraz at 0.75 μg/ml (the EC_90_ of most prochloraz-sensitive isolates). After 10–15 days at 28°C in the dark, cultures growing from the plugs were transferred to new PDA plates containing 0.75 μg/ml prochloraz. This step was repeated until there was no significant difference in the linear growth of fast growing sectors on the PDA plates with or without 0.75 μg/ml prochloraz. To obtain mutants with high resistance to prochloraz, the procedure was also performed with six additional concentrations of prochloraz (3, 5, 6, 10, 15, and 20 μg/ml) (Serfling et al., 2007). The same procedure was used to generate mutants resistant to epoxiconazole with 16, 32, 50, 100, 150, 200, 250, 300, and 350 μg/ml and to difenoconazole with 8, 16, 50, 100, 150, 200, 250, 300, and 350 μg/ml. The derived mutants were further examined for their level and stability of resistance to different azoles as described in the following section.

### Characterization of DMI-Resistant Mutants

#### Resistance Level and Stability

Mycelial plugs taken from the periphery of actively growing colonies were transferred 10 times to fresh, fungicide-free PDA medium. EC_50_ values were determined before the transfer and after the 10th transfer (Pang et al., 2013). Each mutant was evaluated in triplicate, and the experiment was conducted twice.

#### Mycelial Growth and Conidia Production

Culture plugs of the resistant mutants and their parents were incubated on PDA plates at 4, 14, 20, 25, 28, or 37°C with three replicates per temperature; after 4 days colony diameters were measured (Pang et al., 2013). For conidia production, a 4-day-old colony was placed under a black light lamp for 7 days (Fernando et al., 2000). Conidia were harvested by rinsing the sporulating colonies on each plate with 10 ml of distilled water. Conidia in the resulting suspension were quantified with a hemacytometer. Each resistant mutant and parent was represented by three replicate plates, and the experiment was performed twice.

#### Virulence on Chili Pepper

Chili pepper fruit were inoculated with the mutants and their parents as described previously (Than et al., 2008) with some modifications. Briefly, one mycelial plug from a *C. truncatum* culture was placed on each disinfested fruit. Fruit inoculated with PDA plugs without fungus were used as a control. The fruit were then incubated at 28°C with 75% relative humidity and a 12-h light/dark cycle. Each treatment had 10 replicate fruits. After 7 days for inoculation, disease severity was scored on a scale from 0 to 9 as described by Dasgupta (1981) with slight modification. The experiment was conducted twice.

### Cross-Resistance

The EC_50_ values were determined for the mutants and their parents with the three agricultural DMIs, two clinical DMIs, and three non-DMIs listed in Supplementary Table S1 (Russell, 2004; Serfling et al., 2007; Chen et al., 2012). Each treatment had three replicate plates, and the experiment was conducted twice.

### Cloning and Sequencing of the *CtCYP51* Gene

The amplified *CtCYP51* full-length fragments (primers are listed in Supplementary Table S3) were purified using the EasyPure Quick Gel Extraction Kit (TransGen, Beijing, China) and were cloned into the pEASY-T1 simple plasmid (TransGen, Beijing, China) according to the manufacturer’s recommendations. The vector that inserted into *Escherichia coli* strain was sequenced (Sunbiotech Co., Beijing, China) using vector primers M13F (5′-ACTGGCCGTCGTTTTAC-3′) and M13R (5′-GTCCTTTGT CGATACTG-3′) (Pang et al., 2013; Fan et al., 2014). DNA sequences were analyzed with DNAMAN5.2.2.0 (Lynnon Biosoft, Quebec, QC, Canada).

### Quantitative Expression of the *CtCYP51* Gene

For both mutants and their parents, ten mycelial plugs from 4-day-old cultures were added to 60 ml of potato dextrose broth in a 100 ml flask. The flasks were incubated for 24 h on a rotary shaker at 120 rpm. Either prochloraz (0.5 μg/ml), epoxiconazole (30 μg/ml), or difenoconazole (25 μg/ml) were added to the corresponding flasks; other flasks were free of fungicides. After 24 h, mycelia were harvested by vacuum filtration for RNA extraction (Fan et al., 2014). Total RNA was extracted using the SV Total RNA Isolation kit (Promega, Beijing, China), and cDNA was synthesized using the PrimeScrip RT reagent Kit with gDNA Eraser (Takara, Beijing, China) following the manufacturer’s protocol. Three independent experiments were conducted.

Real-time (RT)-PCR was performed with an ABI7500 sequence detection system (Applied Biosystems, United States). Amplifications were conducted using the SYBR Premix Dimer Eraser kit (Takara, Beijing, China). The relative quantities (RQ) of products were calculated using the 2^-ΔΔCt^ method. The β-tublin and glyceraldehyde-3-phosphate dehydrogenase (GAPDH) genes were used as references to normalize the quantification of *CtCYP51* expression (Fan et al., 2014). The thermal cycling conditions and primers are listed in Supplementary Table S3.

### Molecular Docking Analysis

Bioinformatic analysis was used to investigate the molecular docking of different DMI fungicides with CYP51 proteins. The crystal structure of 5EAB, a CYP51 protein from the yeast *Saccharomyces cerevisiae* bound with tebuconazole, was retrieved from the Protein Data Bank and used in the current study. Sequence alignment by DNAMAN software indicated that the CYP51 amino acid residues of *S. cerevisiae* showed about 45% sequence identity with those of *Colletotrichum*. The crystal structure 5EAB was a suitable template for studying the binding conformation of different DMI fungicides with CYP51 proteins. *C. scovillei* is also a representative *Colletotrichum* species in China. Because it was sensitive to all five of the DMIs, the modeling structure of *C. scovillei* was obtained by using the template of 5EAB and the On-line Swiss-model software^2^. The binding cavity was predicted by a ligand mode for tebuconazole complexed in CYP51 by SYBYL 7.3 software. The position of these different amino acids between *C. scovillei* and *C. truncatum* was analyzed in the CYP51 protein; only residues M376 and H373 were in the binding cavity with a distance of less than 10 Å. The Biopolymer-Replace Sequence subset from the SYBYL 7.3 software package was used to produce changes at residues M376 or H373. The Tripos force field with Gasteiger–Marsili charges was used for the energy minimization. Based on the CYP51 structure model of *C. scovillei*, the modeling of *C. truncatum* was built by replacing the amino acid at position 376 from methionine to leucine (M376L), at position 373 from histidine to asparagine (H373N), or at both these two positions (M376L/H373N). The molecular conformations of difenoconazole, tebuconazole, and myclobutanil were constructed by Sketch mode and were optimized using the Tripos force field and Gasteiger–Hückel charge. The Surflex-Dock of SYBYL 7.3 was used for the molecular docking modeling (Jain, 2003). The binding cavity was set as “ligand” and the total score was used to evaluate the binding affinity between ligand and protein (Jain, 1996). All molecular modeling between the putative *C. scovillei* and *C. truncatum* CYP51 proteins with ligand was conducted on the Silicon Graphics^®^ (SGI) Fuel Workstation (Silicon Graphics International Corp., Milpitas, CA, United States).

### Statistical Analysis

Data were analyzed using DPS software ver. 7.05 (Zhejiang University, Hangzhou, China). Means were separated using Duncan’s multiple range test at *P* = 0.05. Cross-resistance between two fungicides was analyzed using Spearman’s rank correlation coefficient with log-transformed EC_50_ values (Chen et al., 2012; Pang et al., 2013).

## Results

### The Sensitivities of *Colletotrichum truncatum* to Five DMI Fungicides

**FIGURE 1:**
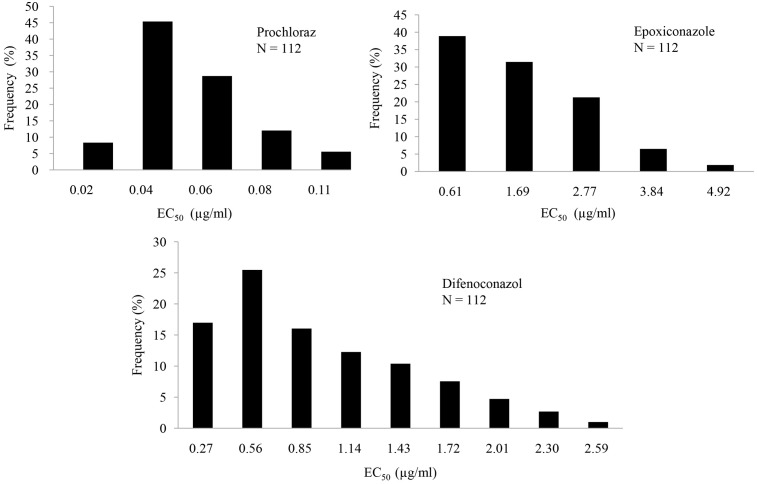
Frequency distributions of effective concentrations for 50% growth inhibition (EC_50_) of prochloraz, epoxiconazole, and difenoconazole against 112 *Colletotrichum truncatum* isolates.

EC_50_ values were determined based on the growth of the 112 isolates on PDA containing different concentrations of five commonly used agricultural DMIs. The EC_50_ value (mean ± SD, μg/ml) was 0.053 ± 0.023 for prochloraz, 1.027 ± 0.644 for difenoconazole, 1.956 ± 0.815 for epoxiconazole, >40 for tebuconazole, and >100 for myclobutanil. The values indicated that *C. truncatum* isolates were quite sensitive to prochloraz, difenoconazole, and epoxiconazole but not to tebuconazole or myclobutanil. These results suggest differential fungicide sensitivity in *C. truncatum*. The distributions of EC_50_ values for prochloraz, epoxiconazole, and difenoconazole were unimodal, thus indicating the absence of azole-resistant subpopulations among the 112 representative isolates (**Figure 1**).

### Generation of DMI-Resistant Mutants

To evaluate the risk and molecular mechanism in developing resistance to these three effective DMIs in *C. truncatum*, mutants were generated by exposing *C. truncatum* cultures to prochloraz, difenoconazole, and epoxiconazole. A total of 11 DMI-resistant mutants were derived from the 112 isolates (**Table 1**). Two mutants were resistant to prochloraz, three were resistant to epoxiconazole, and six were resistant to difenoconazole. These resistant mutants were all derived from different parental isolates, and no mutants were obtained from other wild-type isolates. Mutation frequencies of *C. truncatum* to DMIs were high, and reached values of 2 × 10^-4^ for prochloraz, 6 × 10^-4^ for epoxiconazole, and 1 × 10^-3^ for difenoconazole.

**Table 1:** Resistance factor and resistance stability of DMI-resistant mutants of *Colletotrichum truncatum*.

Fungicide	WT isolate or mutant^a^	Code^b^	EC_50_ ± standard deviation (μg/ml)		RF for EC_50_^c^		FSC^d^	MIC (μg/ml)	RF for MIC^e^
			1st^f^	10th	1st	10th			
Prochloraz	LNXC1-14	PS1	0.06 ± 0.01	0.05 ± 0.01				0.75	
	P15-LNXC1-14	PR1	1.12 ± 0.09	0.75 ± 0.06	20.19	14.92	0.74	5.00	6.67


	LNXC1-15	PS2	0.05 ± 0.01	0.03 ± 0.01				0.75	
	P20-LNXC1-15	PR2	0.30 ± 0.04	0.13 ± 0.02	6.02	4.33	0.72	3.00	4.00


Epoxiconazole	SDWC2-35	ES1	2.86 ± 0.13	2.86 ± 0.32				15.00	


	E200-SDWC2-35	ER1	80.67 ± 10.01	55.51 ± 4.55	28.21	19.41	0.69	400.00	26.67
	GDQY13-52	ES2	2.36 ± 0.22	2.57 ± 0.36				50.00	
	E200-GDQY13-52	ER2	31.29 ± 2.41	23.65 ± 1.84	13.26	9.20	0.69	400.00	8.00
	GDMM9	ES3	3.31 ± 0.40	2.97 ± 0.23				25.00	


	E200-GDMM9	ER3	13.55 ± 1.95	24.22 ± 2.76	4.09	8.15	1.99	300.00	12.00
Difenoconazole	LNXC2-14	DS1	1.70 ± 0.07	1.59 ± 0.04				10.00	


	D350-LNXC2-14	DR1	106.11 ± 9.45	51.85 ± 6.20	62.56	32.61	0.52	500.00	50.00
	GDQY13-66	DS2	1.20 ± 0.09	1.20 ± 0.05				20.00	
	D350-GDQY13-66	DR2	43.38 ± 6.62	28.78 ± 4.36	36.27	24.06	0.66	500.00	25.00
	CQCT12	DS3	1.21 ± 0.04	1.16 ± 0.06				15.00	
	D350-CQCT12	DR3	21.48 ± 1.21	22.56 ± 2.00	17.73	19.42	1.10	200.00	13.33
	HBLF11-2	DS4	1.03 ± 0.01	1.05 ± 0.01				10.00	


	D350-HBLF11-2	DR4	33.84 ± 5.32	42.77 ± 3.68	32.88	40.73	1.24	500.00	50.00
	TJWQ7	DS5	1.37 ± 0.09	1.26 ± 0.05				20.00	
	D350-TJWQ7	DR5	32.21 ± 2.78	22.01 ± 3.12	23.56	17.51	0.74	200.00	10.00
	GDMM4	DS6	1.63 ± 0.25	1.65 ± 0.10				25.00	


	D350-GDMM4	DR6	15.76 ± 2.04	9.96 ± 1.31	9.70	6.04	0.62	200.00	8.00

^a^*WT, wide type. ^b^The first letter refers to the fungicide prochloraz (P), epoxiconazole (E), and difenoconazole (D); the second letter refers to sensitive parental isolates (S) or resistant mutants (R). ^c^RF for EC_*50*_ = resistance factor, the radio of the EC_*50*_ of the fungicide-resistant mutant to that of its wild-type parent. ^d^FSC = factor of sensitivity change, the radio of RF values for EC_*50*_ at the 10th to the 1st transfer. ^e^RF for MIC = resistance factor, the ratio of the minimum inhibitory concentration (MIC value) of the fungicide-resistant mutant to that of its wild-type parent. ^f^Number of generation*.

### Characteristics of DMI-Resistant Mutants

#### Resistance Level and Stability

To determine the fitness of DMI-resistant mutants, the level of DMI resistance of the mutants was measured. RF values (resistance factor, the ratio of the EC_50_ value of a resistant mutant to that of its parent) ranged from 4.09 to 62.56, and RF values for the minimum inhibitory concentration (MIC value) ranged from 4.00 to 50.00 (**Table 1**). After 10 transfers on fungicide-free PDA, the FSC values (factor of sensitivity change, the ratio of RF values at the 10th to the 1st transfer) ranged from 0.52 to 1.99 (**Table 1**). FSC values were > 1 for mutants ER3, DR3 and DR4 indicating that the resistance was relatively stable.

#### Effect of Temperature on Mycelial Growth

For the DMI-resistant mutants and parental isolates of *C. truncatum*, the optimal temperature for mycelial growth was 28°C (**Table 2**). Mutants PR2 and ER2 grew faster than their parental isolates at all five temperatures. Mutants PR1 and ER3 grew faster than their parental isolates at 14°C and 20°C but grew at the same rate or slower than their parental isolates at 25, 28, and 37°C. Mutants DR5 and DR6 grew at the same rate or slower than their parental isolates at 14, 20, 25, and 28°C but grew faster than their parental isolates at 37°C (**Table 2**). In summary, some of the prochloraz and epoxiconazole mutants had an increased tolerance for low temperatures while some of the difenoconazole mutants had an increased tolerance for high temperatures.

**Table 2:** Effect of temperature on mycelial growth of DMI-resistant mutants of *Colletotrichum truncatum* and their wild-type parents on PDA.

Isolate or mutant^b^	Colony diameter (mm)^a^				
	14°C	20°C	25°C	28°C	37°C
PS1	10.00b	20.50c	35.33c	43.50c	10.17b
PR1	12.67a	22.25b	37.33c	44.17c	9.17b
PS2	11.50b	22.50b	42.33b	50.17b	10.67b
PR2	14.33a	25.67a	45.83a	53.50a	13.83a
ES1	23.17a	39.00b	42.50a	51.00a	15.50c
ER1	11.07b	25.83c	29.67c	41.75d	15.33c
ES2	12.83b	25.00c	25.33d	42.75d	8.50d
ER2	22.50a	42.00a	41.50ab	48.75b	22.00a
ES3	12.83b	27.33c	40.15b	45.50c	17.80b
ER3	21.17a	38.00b	40.17b	45.75c	18.00b
DS1	12.83b	24.17d	32.83c	37.50c	11.67c
DR1	10.50cd	23.83d	29.00de	37.33c	5.17f
DS2	18.00a	31.50ab	26.67ef	35.17cd	16.83b
DR2	11.33bc	24.67d	26.83ef	25.67f	9.17d
DS3	18.00a	28.50bc	36.33b	43.50b	15.83b
DR3	10.83cd	20.50e	17.50g	29.00ef	13.00c
DS4	16.50a	30.00ab	43.83a	49.50a	6.00ef
DR4	13.00b	26.00cd	31.00cd	32.00de	7.67de
DS5	16.83a	32.50a	44.67a	47.33a	13.17c
DR5	9.33d	17.33e	25.50f	30.67e	17.33b
DS6	11.67bc	29.83ab	30.17d	41.50b	6.00ef
DR6	10.67cd	24.67d	29.67d	37.33g	22.00a

^a^*Values in a column followed by the same letter are not significantly different (*P* < 0.05). ^b^The first letter refers to fungicide [prochloraz (P), epoxiconazole (E), and difenoconazole (D)], and the second letter refers to the sensitive parental isolates (S) or the resistant mutants (R)*.

#### Mycelial Growth, Conidia Production, and Virulence

For most of the DMI-resistant mutants of *C. truncatum*, colony diameter after 4 days at 28°C was equal to or smaller than that of the parental isolate (*P* < 0.05), except for PR2 and ER2 (**Table 3**). Spore production was significantly lower (*P* < 0.05) for the DMI-resistant mutants than for their parents except for ER3 and DR2 (**Table 3**). Some mutants failed to produce conidia. As indicated by disease scores, virulence was also lower for most mutants than for their parental isolates (**Table 3**). Among the 11 mutants, only PR1 had a higher disease score than its parent (*P* < 0.05). A compound fitness index (CFI) was calculated: CFI = *in vitro* mycelial growth × spore production × disease score. The CFI values were significantly lower for most mutants than for their corresponding parents (**Table 3**).

**Table 3:** Fitness of DMI-sensitive (parental) isolates and DMI-resistant mutants of *Colletotrichum truncatum*.

Isolate or mutant^b^	Fitness^a^			
	Colony diameter(mm)	Sporulation (×10^5^/cm^2^)	Disease score	CFI^c^ (×10^7^)
PS1	43.50c	366.67a	1.00c	157.45b
PR1	44.17c	0.90c	3.67b	1.38c
PS2	50.17b	220.00b	5.67a	605.64a
PR2	53.50a	1.63c	1.00c	0.81c
ES1	51.00a	124.00a	3.67b	22.34a
ER1	41.75d	0b	1.00c	0b
ES2	42.75d	114.67a	5.00a	249.28a
ER2	48.75b	0b	1.33c	0b
ES3	45.50c	128.00a	4.33ab	163.80a
ER3	45.75c	152.00a	1.33c	80.06b
DS1	37.50c	36.60c	5.67bc	88.75b
DR1	37.33c	0d	1.00e	0c
DS2	35.17cd	7.23d	7.00a	17.85c
DR2	25.67f	9.10d	3.67d	15.73c
DS3	43.50b	91.67a	6.33ab	248.43a
DR3	29.00ef	9.20d	1.00e	1.90c
DS4	49.50a	57.67bc	5.00c	191.25b
DR4	32.00de	0d	1.00e	0c
DS5	47.33a	41.67c	7.00a	151.34b
DR5	30.67e	0.43d	1.00e	0.08c
DS6	41.50b	71.00b	7.00a	215.25a
DR6	37.33g	17.67d	1.67e	6.66c

^a^*Values in a column followed by the same letter are not significantly different (*P* < 0.05). ^b^The first letter refers to the fungicide prochloraz (P), epoxiconazole (E), and difenoconazole (D); the second letter refers to sensitive parental isolates (S) or resistant mutants (R). ^c^CFI: compound fitness index, calculated as *in vitro* mycelial growth × spore production × disease score*.

### Cross-Resistance

Among the 11 DMI-resistant mutants of *C. truncatum*, cross-resistance to the agricultural azoles prochloraz, epoxiconazole, and difenoconazole could be observed (**Table 4**). Cross-resistance to other azoles (fluconazole, ketoconazole) could be measured in the 11 DMI-resistant mutants, however, no cross-resistance could be observed with non-DMI fungicides including azoxystrobin, carbendazim, and mancozeb (**Table 4**).

**Table 4:** Cross-resistance among three agricultural DMIs (prochloraz, epoxiconazole, and difenoconazole), two clinical DMIs (fluconazole and ketoconazole), and three non-DMIs (azoxystrobin, carbendazim, and mancozeb).

Isolate/mutant^a^	RF^b^							
	Prochloraz	Epoxiconazole	Difenoconazole	Fluconazole	Ketoconazole	Azoxystrobin	Carbendazim	Mancozeb
PR1/PS1	14.92 ± 1.13	295.07 ± 31.15	212.05 ± 7.57	33.88 ± 2.06	15.75 ± 0.15	1.42 ± 0.31	1.69 ± 0.10	1.13 ± 0.32
PR2/PS2	4.33 ± 0.16	35.22 ± 2.40	28.72 ± 1.13	36.37 ± 4.11	8.29 ± 0.66	0.89 ± 0.13	1.00 ± 0.08	0.85 ± 0.19
ER1/ES1	3.67 ± 0.32	19.41 ± 1.36	15.50 ± 1.79	115.99 ± 9.50	12.39 ± 1.00	1.64 ± 0.12	1.33 ± 0.21	1.68 ± 0.06
ER2/ES2	3.40 ± 0.25	9.20 ± 0.32	6.06 ± 0.47	4.76 ± 0.22	5.57 ± 0.08	0.78 ± 0.04	1.00 ± 0.15	0.75 ± 0.20
ER3/ES3	2.92 ± 0.44	8.15 ± 0.47	6.58 ± 1.15	3.98 ± 0.43	6.92 ± 0.24	0.74 ± 0.15	0.76 ± 0.14	0.70 ± 0.15
DR1/DS1	5.09 ± 1.01	87.12 ± 14.22	32.61 ± 2.01	27.97 ± 4.01	24.44 ± 2.22	1.23 ± 0.20	1.25 ± 0.32	1.82 ± 0.43
DR2/DS2	6.74 ± 0.75	14.40 ± 1.52	24.06 ± 0.90	15.76 ± 0.76	19.43 ± 0.55	0.68 ± 0.34	1.25 ± 0.04	2.21 ± 0.54
DR3/DS3	2.92 ± 0.08	69.49 ± 1.90	19.42 ± 0.56	13.61 ± 1.55	14.09 ± 0.79	1.52 ± 0.46	1.14 ± 0.09	1.47 ± 0.30
DR4/DS4	3.09 ± 0.49	5.53 ± 0.64	40.73 ± 3.30	33.71 ± 2.78	25.42 ± 1.43	0.98 ± 0.08	0.95 ± 0.23	2.40 ± 0.67
DR5/DS5	3.68 ± 0.07	5.39 ± 1.10	17.51 ± 1.22	11.09 ± 2.00	21.80 ± 0.87	1.75 ± 0.11	1.30 ± 0.05	1.83 ± 0.12
DR6/DS6	2.11 ± 0.42	4.84 ± 0.20	3.62 ± 0.24	3.37 ± 0.26	2.25 ± 0.06	1.10 ± 0.05	1.00 ± 0.10	1.54 ± 0.08

^a^*The first letter refers to the fungicide prochloraz (P), epoxiconazole (E), and difenoconazole (D); the second letter refers to sensitive parental isolates (S) or resistant mutants (R). ^b^RF = resistance factor, the ratio of the EC_*50*_ of the fungicide-resistant mutant to that of its wild-type parent*.

### Sequence Analysis and Quantitative Expression of the *CtCYP51* Gene

**FIGURE 2:**
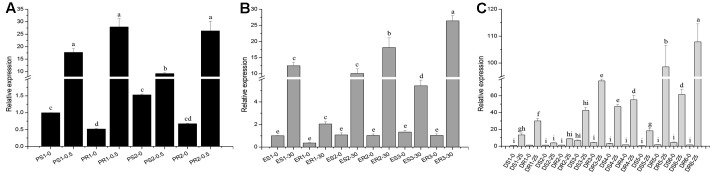
Constitutive and fungicide-induced expression of *CtCYP51* in *Colletotrichum truncatum*. Relative expression of *CtCYP51* was calculated by the 2^-ΔΔCt^ method with the β-tublin and GAPDH genes as references. For the isolate/mutant designations along the *X* axis: the first letter refers to the fungicide prochloraz (P), epoxiconazole (E), or difenoconazole (D); the second letter refers to sensitive parental isolates (S) or resistant mutants (R); the number following the dash is the rate of fungicide application, and 0.5, 30, or 25 after the dash indicate treatment with prochloraz at 0.5 μg/ml, epoxiconazole at 30 μg/ml, or difenoconazole at 25 μg/ml, respectively. Expression in isolates or mutants to **(A)** prochloraz, **(B)** epoxiconazole, or **(C)** difenoconazole was relative to the expression of isolate PS1-0 in **(A)**, ES1-0 in **(B)**, or DS1-0 in **(C)**.

To explore the genetic mechanism of *C. truncatum* resistant to the three effective DMIs, the sequence and expression level of *CtCYP51* were analyzed in this study. The *CtCYP51* gene had a length of 1719 bp, with two introns of 78 and 60 bp, and coded for 526 amino acids. When sequences of *CtCYP51* were compared in DMI-resistant mutants and their sensitive parental isolates, no mutation was detected in any of the mutants. After treatment with prochloraz, *CtCYP51* expression increased by about 17-fold in PS1, 6-fold in PS2, 52-fold in PR1, and 39-fold in PR2 (**Figure 2A**), which was about 3–6 fold higher in prochloraz-treated PR1 and PR2 than in PS1 and PS2. After treatment with epoxiconazole, *CtCYP51* expression increased by about 12-, 9-, and 4-fold in ES1, ES2, and ES3 and by about 6-, 18-, and 26- fold in ER1, ER2, and ER3 (**Figure 2B**). Thus, epoxiconazole treatment resulted in about 2–6-fold higher *CtCYP51* expression in ER2 and ER3 as compared to ES2 and ES3. After treatment with difenoconazole, *CtCYP51* expression increased by about 3- to 35-fold in the six difenoconazole-sensitive isolates but about 7- to 81-fold in the difenoconazole-resistant isolates (**Figure 2C**), i.e., difenoconazole treatment caused 2- to 6-fold up-regulation in *CtCYP51* expression in the difenoconazole-resistant mutants relative to their difenoconazole-sensitive parental isolates.

### Analysis of the Affinity between *Colletotrichum* CYP51 Proteins and DMI Fungicides

**FIGURE 3:**
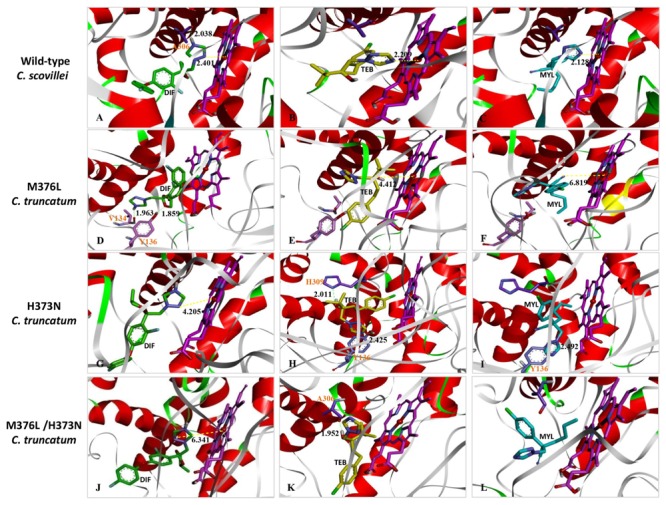
Docking of DMI fungicides in the modeled binding pockets of CYP51 in *C. scovillei* and *C. truncatum*. The CYP51 models have mutations M376L, H373N, or M376L and H373N. The fungicides are difenoconazole (DIF, green), tebuconazole (TEB, yellow), and myclobutanil (MYL, cyan). The structure of *C. scovillei* was modeled with the template of 5EAB from *Saccharomyces cerevisiae* by the On-line Swiss-model software. The structure of *C. truncatum* CYP51 was modeled by mutations at residues M376 (M376L), H373 (H373N), or both M376 and H373 (M376L/H373N) based on the modeling of *C. scovillei*, and the Tripos force field with Gasteiger-Marsili charges was used for the energy minimization. **(A–C)** Represent the binding pockets of the three DMI fungicides in the CYP51 of *C. scovillei*; **(D–L)** represent the binding pockets of the three DMI fungicides in the CYP51 of *C. truncatum* with different mutations (**D–F**: M376L, G-I: H373N, J-L: M376L /H373N). The yellow dash indicates the Fe – N coordinate bond between the heme iron active site and oxygen heterocyclic ring of the DMI fungicide. The green dash represents the hydrogen bond between the amino acid and the fungicide.

Based on the result that *C. truncatum* was only sensitive to prochloraz, epoxiconazole, and difenoconazole, but not to tebuconazole or myclobutanil, the basis for differential DMI sensitivity was investigated. The models were built for the docking of three representative DMI fungicides into *C. scovillei* or *C. truncatum* CYP51 binding pocket (**Figure 3**). The docking scores of these predicted models were shown in **Table 5**. The model for the wild-type *C. scovillei* indicated that the Fe – nitrogen coordinate bond between the heme iron active site and the DMI heterocyclic nitrogen atom was at a distance of 2.401, 2.209, and 2.128 Å for difenoconazole, tebuconazole, and myclobutanil, respectively (**Figures 3A–C**). In addition, there was a hydrogen bond interaction (2.038 Å) between the A306 and –NH group of nitrogen heterocyclic ring of difenoconazole (**Figure 3A**).

**Table 5:** Total scores of three DMI fungicides docked into the CYP51 binding site of *C. scovillei* and *C. truncatum* models (M376L, H373N, M376L /H373N).

Fungicide	Docking score			
	Wild-type^a^ *C. scovillei*	M376L^b^ *C. truncatum*	H373N^b^ *C. truncatum*	M376L /H373N^b^ *C. truncatum*
Difenoconazole	8.18	7.45	6.75	6.00
Tebuconazole	8.38	5.27	4.39	3.80
Myclobutanil	8.00	5.06	3.76	3.49

^a^*The modeling structure of *C. scovillei* was obtained with the template of 5EAB by the On-line Swiss-model software; ^b^The structure of *C. truncatum* was modeled with a mutation at residues M376 (M376L), H373 (H373N), or both M376 and H373 (M376L/H373N) based on the modeling of *C. scovillei*, and the Tripos force field with Gasteiger-Marsili charges was used for the energy minimization*.

The different CYP51 amino acids between *C. scovillei* and *C. truncatum* were analyzed; only residues at position 376 and 373 were in the CYP51 binding cavity that bound to DMIs. The residues M376 and H373 in the structure model of *C. scovillei* were substituted to the corresponding amino acid L376 and N373 for the modeling of *C. truncatum* using the SYBYL 7.3 software, respectively. The amino acid change M376L changed the conformation of these three DMI fungicides in the CYP51 binding cavity and thereby led to the loss of the Fe – N coordinate bond (**Figures 3D–F**). For difenoconazole, the replacement of methionine by leucine generated two strong hydrogen bonds between difenoconazole with V134 (1.963 Å) and T136 (1.859 Å), and the docking score remained high at 7.45 (**Figure 3D** and **Table 5**). However, there was only a weak Fe – O coordinate bond (4.412 Å) between the heme iron active site with the O atom of tebuconazole, and a weaker Fe – N coordinate bond (6.819 Å) with the N atom of cyano group of myclobutanil, which decreased the docking score decreased from about 8 to 5 (**Figures 3E,F** and **Table 5**). The H373N mutation changed the Fe – N coordinate bond between difenoconazole and the heme iron from 2.401 to 4.205 Å (**Figure 3G**). As a result, the docking score changed to 8.18 for H373 and to 6.75 for N373 (**Table 5**). Although two new hydrogen bonds formed between tebuconazole and CYP51 amino acids, 2.011 Å with H309 and 2.425 Å with Y136, the mutation H373N eliminated the strong Fe – N coordinate bond (2.209 Å) between tebuconazole and the heme iron, which decreased the docking score from 8.38 to 4.39 for tebuconazole (**Figure 3H** and **Table 5**). The changes in the interaction for myclobutanil were similar to those for tebuconazole (**Figure 3I**), and decreased the docking score from 8.00 to 3.76 (**Table 5**).

The double mutations at positions 376 and 373 in the CYP51 of *C. truncatum* weakened the Fe – N coordinate bond interaction for difenoconazole from 2.401 to 6.341 Å and decreased the docking score from 8.18 to 6.00 (**Figure 3J**). For tebuconazole and myclobutanil, however, the double mutation M376L/H373N eliminated the Fe – N coordinate bond interactions between the fungicide and the heme iron active site, which caused the docking score decrease to 3.80 for tebuconazole and 3.49 for myclobutanil (**Figures 3K,L** and **Table 5**). The changes in the docking score were consistent with the results of the fungicide sensitivity assays, in which *C. truncatum* showed strong insensitivity to tebuconazole and myclobutanil but high sensitivity to difenoconazole.

## Discussion

DMI inhibitors that have been used as fungicides include imidazoles and triazoles. Although these two groups of fungicides have a similar mode of action, differential sensitivity was observed to different DMIs in *C. truncatum*. The ranking of sensitivity was myclobutanil < tebuconazole < epoxiconazole < difenoconazole < prochloraz, i.e., the isolates were most sensitive to the imidazole prochloraz and were least sensitive to the triazoles myclobutanil and tebuconazole. The EC_50_ values were > 40 μg/ml for tebuconazole, and >100 μg/ml for myclobutanil, indicating that they had almost no effect on *C. truncatum*. These results suggest that the triazole fungicides tebuconazole and myclobutanil should not be used to control *C. truncatum* in pepper fields in China. In contrast, a previous study found that two *C. cereale* populations were quite sensitive to myclobutanil and tebuconazole (Wong and Midland, 2007). The sensitivity of different *Colletotrichum* species to various DMIs needs to be explored in the future.

Because no DMI-resistant subpopulation was found in the field, the data for epoxiconazole, difenoconazole, and prochloraz sensitivity may provide a reference for monitoring sensitivity changes in *C. truncatum* populations to these fungicides in China. For the *C. truncatum* mutants that became resistant to prochloraz, epoxiconazole, and difenoconazole after exposure to these fungicides in the laboratory, mutation frequencies were high. Similar frequencies were reported for the development of resistance to prochloraz and tebuconazole in iaolates of the *C. gloeosporioides* species complex from strawberry and grape (Xu et al., 2014). All DMI-resistant mutants in the current study exhibited impaired fitness, which was consistent with reports for DMI-resistant mutants of other filamentous fungi, including *Monilinia fructicola* mutants resistant to SYP-Z048 (Chen et al., 2012), and *Aspergillus nidulans* mutants resistant to imazalil (Vantuyl, 1977). If laboratory mutants have a high level of fitness, this suggests that resistance can occur in field populations. In the current study, however, the reduced fitness of the resistant mutants suggests a limited probability of resistance in field populations of *C. truncatum*.

The *C. truncatum* mutants that were resistant to agricultural DMIs were also less sensitive than their parental isolates to the tested medical azoles. Likewise, tebuconazole-adapted *C. graminicola* strains were less sensitive than parental isolates to clinical azoles in a previous study (Serfling et al., 2007). Cross-resistance in azoles has been observed in yeast isolates obtained from the oropharynx of human patients and from the environment (Muller et al., 2007). That cross-resistance was not found between DMI and non-DMI fungicide in the current study, which is not surprising because the two groups of fungicides have very different modes of action.

Amino acid alterations resulting from mutation in the *CYP51* gene and increased expression of the *CYP51* gene are common mechanisms of resistance to azoles in fungi (Wyand and Brown, 2005; Ma et al., 2006; Nina et al., 2015). In this study, no mutation was detected in *CYP51* in any of the 11 *C. truncatum* mutants. The expression of *CYP51* in all of the parental and resistant isolates was increased by treatment with the three azole fungicides. This up-regulation caused by DMI treatment was about 2- to 6-times higher in the mutants than in their corresponding parental isolates for 10 of the 11 mutants, except for ER1. This indicated that induced expression of *CtCYP51* could play a role in the resistance to DMIs. Resistance to DMIs in *C. truncatum* might involve the overexpression of ATP binding cassette (ABC) transporters or major facilitator superfamily (MFS) transporters (; Hayashi et al., 2002; Kretschmer et al., 2009), and this possibility should be tested in the future.

Molecular docking was used to explore why *C. truncatum* sensitivity differs among DMI fungicides, and our analysis indicated that only residues M376 and H373 were in the CYP51 binding cavity that bound to DMIs. Therefore, the residues M376 and H373 in the *C. scovillei* CYP51 were changed *in silico* to corresponding amino acids in *C. truncatum*. There was little change in the docking score for difenoconazole, but the docking scores for tebuconazole and myclobutanil were significantly decreased when these two points changed to the corresponding amino acid in *C. truncatum*. The changes in the docking score were consistent with the results of the fungicide sensitivity assays, which showed that *C. truncatum* was quite insensitivity to tebuconazole and myclobutanil. Other studies have reported that some mutations in fungal binding sites can have different effects on fungicides with the same mode of action. For example, the Y145F mutation in *C. neoformans* CYP51 and Y136F mutation in *Histoplasma capsulatum* CYP51 both cause resistance to the short-tailed triazoles fluconazole and voriconazole but not to the long-tailed triazoles posaconazole or itraconazole (Wheat et al., 2006; Sionov et al., 2012). Another study reported that zoxamide sensitivity can be affected by changes at E198 but the mutation E198V had no effect on the sensitivity of *Botrytis cinerea* to carbendazim (Cai et al., 2015). The current study indicated that the M376L/H373N changes altered the conformation of DMIs in the binding pocket, and prevented the formation of Fe – O coordinate bond between the heme iron active site and tebuconazole or myclobutanil in *C. truncatum* (**Figures 3G–L**); this caused the docking scores to sharply decline and explained why this mutation resulted in resistance only to tebuconazole and myclobutanil. In conclusion, this study investigated the differences in the sensitivity of *C. truncatum* to different DMI fungicides. Molecular docking models were used to predict how the mutations in the CYP51 binding pocket of *C. truncatum* alter the binding of DMIs. The results increase our understanding of DMI fungicides and may help in the development of more effective fungicides for disease management.

## Author Contributions

XL and CZ designed the experiments. CZ, WW, and MI performed the *in vitro* experiments. CZ, YD, and WW performed the molecular experiments. HD and CZ conducted the molecular docking. YD collected isolates. CZ and JH participated in data analysis and CZ interpreted the results. XL, CZ, and HD wrote the paper. All authors gave final approval for publication.

## Conflict of Interest Statement

The authors declare that the research was conducted in the absence of any commercial or financial relationships that could be construed as a potential conflict of interest.

## Footnotes

1 http://www.chinapesticide.gov.cn/

2 https://www.swissmodel.expasy.org/
