# Supplementary material for: Assessing the Risk for Resistance and Elucidating the Genetics of Colletotrichum truncatum That Is Only Sensitive to Some DMI Fungicides
Source: Front Microbiol. 2017 Sep 15;8:1779. doi: 10.3389/fmicb.2017.01779 (PMC5609536; doi:10.3389/fmicb.2017.01779)
Supplement: Supplementary file 1 [file Table_1.DOCX]

Table S1. Concentrations used to determine the sensitivity of *Colletotrichum truncatum* field isolates and DMI-resistant mutants to various fungicides.

| Fungicide | Active ingredients (a.i.) and distributor^a^ | Fungicide concentration (μg/ml) in agar medium | |
| --- | --- | --- | --- |
|  |  | Field isolate | Azole-resistant mutant^b^ |
| Prochloraz | 97%, Qingfeng Pesticide & Chemicals Co., Hangzhou | 0, 0.10, 0.25, 0.50, 0.75, 1.50 | 0, 0.1, 0.25, 0.5, 1.5, 3 |
| Epoxiconazole | 97.8%; Fengdeng Pesticide Co., Jiangsu | 0, 0.125, 0.25, 1.00, 2.50, 5.00, 8.00 | 0, 1, 2.5, 10, 25, 50, 100, 150 |
| Difenoconazole | 98%; Yulong Chemical Industrial Co., Hangzhou | 0, 0.10, 0.25, 0.50, 1.00, 2.50, 5.00 | 0, 1, 5, 10, 25, 100, 150 |
| Tebuconazole | 98%; Fengdeng Pesticide Co., Jiangsu | 0, 0.50, 1.00, 2.50, 5.00, 10.00, 25.00, 40.00 | - |
| Myclobutanil | 98%; Yulong Chemical Industrial Co., Hangzhou | 0, 2.50, 5.00, 10.00, 25.00, 50.00, 100.00 | - |
| Fluconazole | analytical standards; Sigma-Aldrich, Shanghai | 0, 25, 50, 100, 200, 300 | 0, 50, 100, 200, 300, 400 |
| Ketoconazole | analytical standards; Sigma-Aldrich | 0, 1, 2.5, 10, 40, 100 | 0, 2.5, 10, 40, 100, 200 |
| Azoxystrobin | 95%; Syngenta Biotechnology, Shanghai | 0, 0.05, 0.25, 1, 5, 25,50 | 0, 0.05, 0.25, 1, 5, 25, 50 |
| Carbendazim | 98%; Chunguang Pesticide & Chemicals Co., Henan | 0, 0.025, 0.05, 0.1, 0.2, 0.4 | 0, 0.025, 0.05, 0.1, 0.2, 0.4 |
| Mancozeb | 98.5%; Hesen Chemical Industrial Co., Hebei | 0, 5, 10, 15, 20, 30, 40 | 0, 5, 10, 15, 20, 30, 40 |

^a^All companies were in China.

^b^The mutants were selected in the laboratory by exposing them to azoles fungicides.
