# Supplementary material for: Assessing the Risk for Resistance and Elucidating the Genetics of Colletotrichum truncatum That Is Only Sensitive to Some DMI Fungicides
Source: Front Microbiol. 2017 Sep 15;8:1779. doi: 10.3389/fmicb.2017.01779 (PMC5609536; doi:10.3389/fmicb.2017.01779)
Supplement: Supplementary file 2 [file Table_2.DOCX]

Table S2. *Colletotrichum truncatum* isolates collected from pepper in China.

| Location (city, province) | Designation | Host tissue | Year | Number of isolates |
| --- | --- | --- | --- | --- |
| Qingyuan, Guangdong | GDQY | Fruit | 2013 | 15 |
| Maoming, Guangdong | GDMM | Fruit | 2013 | 9 |
| Yichun, Jiangxi | JXYC | Leaf | 2011 | 9 |
| Chongqing | CQCT | Fruit | 2013 | 8 |
| Wuhan, Hubei | HBWH | Fruit | 2013 | 11 |
| Fengxiang, Shanxi | SXFX | Fruit | 2011 | 7 |
| Wucheng, Shandong | SDWC | Fruit | 2011 | 10 |
| Laiyang, Shandong | SDLY | Fruit | 2011 | 4 |
| Wuqing, Tianjin | TJWQ | Fruit | 2012 | 10 |
| Langfang, Hebei | HBLF | Fruit | 2011 | 8 |
| Shunyi, Beijing | BJSY | Fruit | 2011 | 10 |
| Xingcheng, Liaoning | LNXC | Fruit | 2012 | 7 |
| Changchun, Jilin | JLCC | Fruit | 2012 | 4 |
| Total |  |  |  | 112 |
