# Supplementary material for: Assessing the Risk for Resistance and Elucidating the Genetics of Colletotrichum truncatum That Is Only Sensitive to Some DMI Fungicides
Source: Front Microbiol. 2017 Sep 15;8:1779. doi: 10.3389/fmicb.2017.01779 (PMC5609536; doi:10.3389/fmicb.2017.01779)
Supplement: Supplementary file 3 [file Table_3.DOCX]

Table S3. Primers used in polymerase chain reaction (PCR) for target genes.

| Primer | Sequence (5’-3’) | Application |
| --- | --- | --- |
| Ct-CYP51-F2 | TCACATACGGCAAGAACCCT | Real-time PCR of *CtCYP51* ^a^  Real-time PCR of β-*tublin*  Real-time PCR of GAPDH |
| Ct-CYP51-R2 | TAGATCTCTTCGGCGGACAC |  |
|  |  |  |
| Ct-Tublin-F | GTCCCACGCGTTTTAAGACA |  |
| Ct-Tublin-R | ACCCTCAGTGTAGTGACCCT |  |
|  |  |  |
| Ct-GAPDH-F | ACGACCCCTTCATTGAGACC |  |
| Ct-GAPDH-R | TTGACTTTCTGCCTCACGTC |  |

^a^Reaction parameters for real-time PCR were as follows: 95°C for 30 s; 40 cycles at 95°C for 5 s, 60°C for 30 s, and 72°C for 34 s; and a final dissociation stage at 95°C for 15 s, 60°C for 60 s, 95°C for 15 s, and 60°C for 15 s.
